# Genome-Wide Association Study Identified Copy Number Variants Important for Appendicular Lean Mass

**DOI:** 10.1371/journal.pone.0089776

**Published:** 2014-03-13

**Authors:** Shu Ran, Yong-Jun Liu, Lei Zhang, Yufang Pei, Tie-Lin Yang, Rong Hai, Ying-Ying Han, Yong Lin, Qing Tian, Hong-Wen Deng

**Affiliations:** 1 Center of System Biomedical Sciences, University of Shanghai for Science and Technology, Shanghai, People’s Republic of China; 2 Key Laboratory of Biomedical Information Engineering of the Ministry of Education, Institute of Molecular Genetics, School of Life Science and Technology, Xi’an Jiaotong University, Xi’an, People’s Republic of China; 3 Geriatrics Health Clinic of Inner Mongolia People’s Hospital, Inner Mongolia, People’s Republic of China; 4 Department of Biostatistics and Bioinformatics, Tulane University, New Orleans, Louisiana, United States of America; Wake Forest University Health Sciences, United States of America

## Abstract

Skeletal muscle is a major component of the human body. Age-related loss of muscle mass and function contributes to some public health problems such as sarcopenia and osteoporosis. Skeletal muscle, mainly composed of appendicular lean mass (ALM), is a heritable trait. Copy number variation (CNV) is a common type of human genome variant which may play an important role in the etiology of many human diseases. In this study, we performed genome-wide association analyses of CNV for ALM in 2,286 Caucasian subjects. We then replicated the major findings in 1,627 Chinese subjects. Two CNVs, CNV1191 and CNV2580, were detected to be associated with ALM (*p* = 2.26×10^−2^ and 3.34×10^−3^, respectively). In the Chinese replication sample, the two CNVs achieved *p-*values of 3.26×10^−2^ and 0.107, respectively. CNV1191 covers a gene, GTPase of the immunity-associated protein family (GIMAP1), which is important for skeletal muscle cell survival/death in humans. CNV2580 is located in the Serine hydrolase-like protein (SERHL) gene, which plays an important role in normal peroxisome function and skeletal muscle growth in response to mechanical stimuli. In summary, our study suggested two novel CNVs and the related genes that may contribute to variation in ALM.

## Introduction

Loss and function impairment of skeletal muscle, especially in the elderly, are related to a number of public health problems (such as sarcopenia, osteoporosis) and increased mortality [1], [2]. Whole lean body mass (LBM) is composed of skeletal muscle (∼60%), viscera, and some other connective tissues. Appendicular lean mass (ALM) is sum of skeletal muscle mass in arms and legs which is the primary portion of skeletal muscle involved in ambulation and physical activities. ALM is considered to be an ideal measure for skeletal muscle mass [3], [4], [5], [6]. ALM can be measured accurately by dual energy X-ray absorptiometry (DXA).

Skeletal muscle is under strong genetic control, with heritability estimates of 30–85% for muscle strength and 50–80% for muscle mass [7], [8]. Genome wide association studies have identified a number of variants that may account for variation in ALM [9], [10]. However, collectively, the identified loci/genes/variants only explain a small fraction of genetic variation in ALM, and the majority of the genetic determination remains to be revealed. Traditional association studies have focused on single nucleotide polymorphisms (SNPs). Studies on other types of genetic variants, which may account for the “missing” heritability, have been relatively rare.

Recent studies have shown that copy number variation (CNV) plays an important role in human diseases, such as schizophrenia [11], [12], Parkinson’s disease [13], and autism [14]. CNV is a common type of genomic variability with the size of DNA fragments ranging from one kilobase to several megabases and presents at variable copy numbers in comparison with reference genome [15]. CNV may influence gene expression, phenotypic variation and adaptation by disrupting coding or altering gene dosage [16], [17], [18], [19]. Furthermore, it may affect gene expression indirectly through position effects, predispose to deleterious genetic changes, or provide substrates for chromosome change in evolution [15], [20], [21], [22]. A recent GWAS of CNVs in Chinese identified the gremlin1 gene that was associated with LBM variation [23]. However, to date, no study has been performed to investigate whether CNVs contribute to ALM in other ethnic groups such as Caucasians.

In this study, we performed a CNV-based GWAS to identify genetic loci influencing variation in ALM in 2,286 Caucasian subjects. Follow-up replication analyses were performed in a Chinese population consists of 1,627 subjects.

## Materials and Methods

### Ethics Statement

The study was approved by Institutional Review Boards of Creighton University, University of Missouri-Kansas City, Hunan Normal University of China and Xi’an Jiaotong University of China. Signed informed-consent documents were obtained from all study participants before they entered the study.

### Subjects

The discovery sample consisted of 2,286 unrelated Caucasian subjects that were of European origin recruited in Midwestern US (Kansas City, Missouri and Omaha, Nebraska). The inclusion and exclusion criteria were described in our previous publications [24].

Replication sample is an independent Chinese sample containing 1,627 unrelated subjects. All subjects were recruited from the cities of Xi’an and Changsha and their neighboring areas in China.

### Phenotyping

Anthropometric measures and a structured questionnaire covering lifestyle, diet, family information, medical history, etc. were obtained for all the study subjects. ALM and fat body mass (FBM) were measured using a dual-energy X-ray absorptiometry scanner Hologic QDR 4500 W (Hologic Inc., Bedford, MA, USA), for the all study samples. ALM (kg) was calculated as the sum of lean soft tissue (nonfat, non-bone) mass in the arms and legs. Weight was measured in light indoor clothing, using a calibrated balance beam scale, and height was measured as without shoes using a calibrated stadiometer.

### Genotyping

Genomic DNA was extracted from peripheral blood leukocytes using standard protocols. Genome-Wide Human SNP Array 6.0 (Affymetrix, Santa Clara, CA, USA), which includes 906,600 SNPs and 940,000 copy number probes, was used to genotype each subject from the discovery sample, according to the Affymetrix protocol. Briefly, approximately 250 ng of genomic DNA was digested with restriction enzyme NspI or StyI. Digested DNA was adaptor-ligated and PCR-amplified for each sample. Fragment PCR products were then labeled with biotin, denatured, and hybridized to the arrays. Arrays were then washed and stained using Phycoerythrin on Affymetrix Fluidics Station, and scanned using the GeneChip Scanner 3000 7 G to quantitate fluorescence intensities. Data management and analyses were conducted using the Genotyping Command Console Software. For sample quality control (QC), a contrast QC threshold was set at a default value of greater than 0.4. The final average contrast QC across the entire sample reached a high level of 2.76 for our Caucasian cohort and 2.62 for our Chinese cohort.

### Copy Number Analysis

Common CNVs were identified using the CANARY algorithm implemented in the Birdsuite software [25], which utilized a previously defined copy number polymorphism (CNP, namely CNV with frequency greater than 1%) map based on HapMap samples [26]. In total, 1,216 CNPs were genotyped for the subjects of the discovery sample and 1280 CNPs in the replication sample, respectively.

### QC

We conducted QC filtering both at the sample level and the CNV level, according to the previously reported methods [27].

First, for the sample level QC, we used three quality metrics reported by the Birdseye method to evaluate the initial 2,286 subjects for quality in copy number genotyping. The following procedures were adopted: 1) we removed any sample that was greater or less than three standard deviations (SD) from the average estimate of copy number, which was approximate two copies at genome-wide level; 2) we calculated the variability in copy number and SNP probe intensities with each standardized per chromosome. We removed any sample with more than three SD than these estimates on the average genome-wide level; 3) we removed any sample in which more than two chromosomes failed any of these three metrics, i.e. more than three SD in estimated copy number or excessive CNV or SNP variability for chromosome. According to above criteria, 71 subjects were discarded. The copy numbers of the remaining 2,215 subjects were successfully genotyped using the CANARY software.

Second, we conducted QC filtering at the CNV level. Out of the initially called CNVs, we excluded those with uncertain or missing copy call of >5% or with a minor variant frequency of <1%. We discarded the CNVs with allele frequency of <1%. With the above QC criteria, a total of 410 CNVs remained in the subsequent analyses for the Caucasian sample.

### Statistical Analyses

Association analyses of CNV were performed using a linear regression model in R package “glm” [28]. For both the initial GWAS and subsequent replication studies, stepwise regression was performed to screen the effects of covariates on ALM variation. Age, sex, height, and FBM were significant effectors (*p*<0.05) and raw ALM values were adjusted for these factors. We adjusted for covariates by a 2-stage procedure where the outcomes were regressed on covariates only, and then the resulting residuals were regressed on CNVs. To correct for the effect of potential population stratification, we conducted a principal component analysis on genome-wide SNP data with EIGENSTRAT [29] and included the top ten principal components as covariates. Fisher’s method [30] was used to combine the *p*-values from the discovery sample and replication sample.

## Results

The basic characteristics of the subjects used in both discovery and replication samples are summarized in Table 1.

**Table 1:** Basic characteristics of the study subjects.

	Discovery Sample (Caucasian)			Replication Sample (Chinese)		
	Total	Male	Female	Total	Male	Female
No. of subjects	2,286	558	1,728	1,627	802	825
Age	51.37 (13.75)	50.71 (16.05)	51.59 (12.92)	34.49 (13.24)	31.43 (7.97)	37.46 (13.77)
Height (cm)	164.81 (43.03)	171.66 (70.64)	162.61 (28.65)	164.25 (8.16)	170.27 (5.96)	158.38 (5.22)
Weight (kg)	73.86 (42.67)	83.23 (67.08)	70.83 (30.33)	65.72 (9.61)	65.74 (9.64)	54.63 (8.09)
FBM (kg)	19.34 (5.65)	20.67 (9.30)	20.92 (13.20)	14.01 (2.54)	11.86 (5.10)	16.13 (4.90)
ALM (kg)	22.58 (2.68)	29.92 (4.84)	20.22 (3.54)	19.79 (1.94)	23.95 (3.20)	15.74 (2.10)

Note: The numbers within parentheses are standard deviation (SD).

In the discovery sample, 20 CNVs showed evidence for association with ALM at a *p* value of 0.05 (Table 2). CNV1191 and CNV2580 were replicated in the Chinese sample. The *p* values of CNV1191 in the discovery and replication samples were 2.26×10^−2^ and 3.26×10^−2^, respectively, and *p* values of CNV2580 in the discovery and replication samples were 3.34×10^−3^ and 0.107, respectively (Table 2). The combined *p* values of the two CNVs were 6.05×10^−3^ and 3.27×10^−3^, respectively.

**Table 2:** CNVs achieved a *p* value of 0.05 or less in the discovery and replication samples.

CNV	Chr	Start	End	*p*-value^1^	*p*-value^2^
CNV2563	22	23,993,985	24,248,712	1.70×10^−5^	0.810
CNV11821	11	5,228,247	5,230,232	4.66×10^−5^	0.995
CNV148	1	195,089,940	195,168,372	2.32×10^−4^	0.864
CNV160	1	213,560,092	213,565,727	7.34×10^−4^	0.614
CNV1610	10	58,880,511	58,880,997	1.50×10^−3^	0.698
CNV2057	15	19,803,370	20,089,386	1.63×10^−3^	0.173
CNV11449	8	36,194,697	36,197,883	2.09×10^−3^	0.731
**CNV2580**	**22**	**41,234,550**	**41,276,824**	**3.34×10** ^−**3**^	**0.107**
CNV575	4	34,455,420	34,500,578	3.71×10^−3^	0.485
CNV2546	22	20,055,998	20,175,294	5.29×10^−3^	0.647
CNV2694	23	139,324,076	139,328,860	2.16×10^−2^	0.963
**CNV1191**	**7**	**149,916,734**	**149,932,502**	**2.26×10** ^−**2**^	**3.26×10** ^−**2**^
CNV770	5	176,44,656	17,698,273	2.61×10^−2^	0.810
CNV1004	6	126,225,385	126,228,469	2.89×10^−2^	0.659
CNV200	2	24,460,486	24,464,632	2.97×10^−2^	0.706
CNV825	5	86,151,134	86,154,902	3.12×10^−2^	0.463
CNV2529	21	43,794,765	43,797,240	3.16×10^−2^	0.542
CNV2417	19	47,986,230	48,149,894	3.18×10^−2^	0.695
CNV1282	8	24,201,375	24,207,011	4.01×10^−2^	0.311
CNV2430	19	56,834,427	56,840,009	4.66×10^−2^	0.721

Notes:

1 in discovery samples.

2 in replication samples.

Chr: chromosome.

We further tested association between normal (CN = 2) and deletion (CN = 0, 1) groups, and between normal and duplication (CN = 3, 4) groups, separately. The results showed that while the direction of effect of CNV2580 was consistent in discovery and replication samples, it was not the case for CNV1191 (Table 3). However, both CNVs remained to be significant in the combined analyses.

**Table 3:** Association of normal_deletion and normal_duplication with CNVs.

	pair	*β*	SE	*p*-value	Combined*p*-value
CNV2580					
Caucasian	normal_deletion	−5.53×10^−3^	71.82	0.94	0.99
	normal_duplication	−4.16×10^−2^	15.59	7.71×10^−3^	6.84×10^−3^
Chinese	normal_deletion	−9.27×10^−3^	152.84	0.95	
	normal_duplication	−4.40×10^−2^	27.23	0.11	
CNV1191					
Caucasian	normal_deletion	−1.20×10^−2^	10.43	0.25	0.04
	normal_duplication	−21.3×10^−2^	72.84	3.46×10^−3^	0.02
Chinese	normal_deletion	4.70×10^−2^	21.88	0.03	
	normal_duplication	9.84×10^−3^	94.49	0.92	

Notes:

*β*, the standardized regression coefficient, was estimated in kilograms for ALM.

SE: Standard error.

In addition to the 2-step adjustment procedure for covariates aforementioned, we performed association analyses where CNVs and covariates were included in a single model. The results were quite similar to those of the 2-step procedure (Table S1).

**Figure 1:**
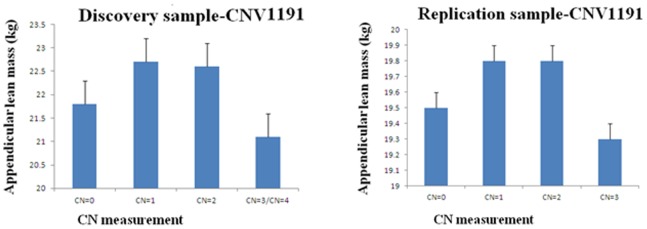
ALM in groups with different copy number (CN) of CNV1191 in the discovery and replication samples.

According to the UCSC Genome Browser on Human February 2009 (GRCh37/hg19) Assembly, CNV1191 is located at the chromosome region 7q36.1 with physical position ranging from 149,916,734 bp to 149,932,502 bp, within the gene GTPase IMAP family member 1 (GIMAP1). The number of carriers with CN = 0, 1, 2, 3 and 4 was 126, 855, 1273, 28 and 4, respectively, in the discovery sample. Due to the limited number of subjects with CN = 4, we merged CN = 3 and CN = 4 into a single group. The number of carriers with CN = 0, 1, 2, 3 was 42, 465, 1093 and 27, respectively, in the replication sample. In the discovery sample, carriers with CN = 1 and CN = 2 had higher ALM (22.7 kg and 22.6 kg) and carriers with CN = 3 had the lowest ALM (21.1 kg) (Figure 1). Consistently, in the replication sample, carriers with CN = 1 and CN = 2 had higher ALM (19.8 kg) and carriers with CN = 3 had the lowest ALM (19.3 kg) (Figure 1).

**Figure 2:**
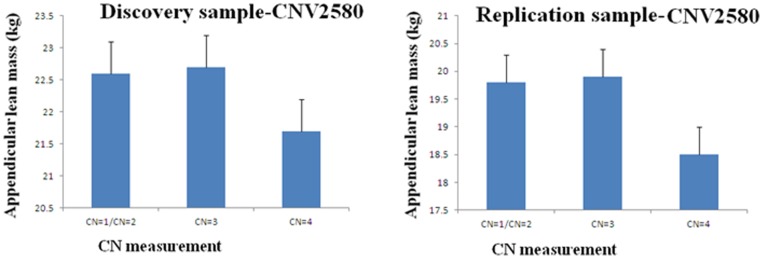
ALM in groups with different CN of CNV2580 in the discovery and replication samples.

CNV2580 is located at the chromosome region 22q13.2 with physical position ranging from 41,234,550 bp to 41,276,824 bp, within the gene serine hydrolase-like protein (SERHL). The number of carriers with CN = 1, 2, 3, and 4 was 11, 1763, 455, and 57, respectively, in the discovery sample, and was 5, 1257, 314 and 50, respectively, in the replication sample. Due to the limited number of subjects with CN = 1, we merged CN = 1 and CN = 2 into a single group. In the discovery sample, carriers with CN = 2 and CN = 3 had higher ALM (22.6 kg and 22.7 kg, respectively) and carrier with CN = 4 had the lowest ALM (21.7 kg) (Figure 2), with the estimated *β* to be −5.24×10^−2^ (ALM in kg) for each copy number. Consistently, in the replication sample, carriers with CN = 2 and CN = 3 had ALM of 19.8 kg and19.9 kg, respectively, and carrier with CN = 4 had ALM of 18.5 kg (Figure 2), with the estimated *β* to be −4.34×10^−2^ (ALM in kg) for each copy number.

Table 4 lists the proportion of subjects for each copy of CNV2580. The table also includes theoretical proportion calculated based on empirical CN frequencies and random mating assumption. Goodness-of-fit (GOF) test showed that empirical distribution did not deviate from the theoretical distribution (*p* = 0.22 for both populations).

**Table 4:** The proportion of the subjects in each CN category of CNV2580.

	Caucasian		Chinese	
	Theoretical	Actual	Theoretical	Actual
CN = 0	1.16×10^−5^	0	4.72×10^−6^	0
CN = 1	4.00×10^−3^	5.00×10^−3^	3.00×10^−3^	3.00×10^−3^
CN = 2	0.76	0.77	0.76	0.77
CN = 3	0.22	0.20	0.22	0.19
CN = 4	0.02	0.02	0.02	0.03
GOF	0.22		0.22	

Note:

GOF: Goodness-of-fit.

There are two SNPs that are located in the region of CNV1191 and eight SNPs outside the CNV1191 boundaries but inside the gene of GIMAP1. None of these ten SNPs was significantly associated with ALM in the discovery sample, but rs11769150 was associated with ALM in the replication sample with *p*-value of 0.02 (Table 5).

**Table 5:** SNPs located in the two CNVs regions or outside the two CNVs boundaries and their association signals with ALM.

CNV region	Chr	SNP	Position	Discovery sample			Replication sample		
				Allele	MAF	*p*-value	Allele	MAF	*p*-value
CNV1191	7	rs6969250	149,923,478	C/T	0.23	NA	C/T	0.17	NA
	7	rs11984138	149,931,668	G/A	0.24	0.77	G/A	0.33	0.9
	7	rs10271154	150,413,004	C/T	0.25	0.82	C/T	0.37	0.46
	7	rs4725388	150,413,550	G/A	0.28	NA	G/A	0.55	NA
	7	rs13239507	150,415,340	T/C	0.28	NA	T/C	0.42	NA
	7	rs6464126	150,416,254	C/T	0.28	NA	C/T	0.42	NA
	7	rs11769150	150,416,686	C/T	0.18	0.82	C/T	0.14	0.02
	7	rs10270107	150,417,979	G/A	0.26	NA	G/A	0.35	NA
	7	rs6957724	150,419,237	C/T	0.24	NA	C/T	0.12	NA
	7	rs11773852	150,421,052	C/T	0.25	0.63	C/T	0.22	0.98
CNV2580	22	rs4820470	41,236,248	A/G	0.15	NA	A/G	0.05	-
	22	rs12485160	41,237,563	C/T	0.13	NA	C/T	0.11	NA
	22	rs5758786	41,256,609	A/A	0	–	A/G	0.02	–
	22	rs4822160	41,262,261	A/G	0.20	0.56	A/G	0.19	0.99
	22	rs9614207	42,898,768	A/G	0.42	NA	A/G	0.23	NA
	22	rs9614208	42,899,411	G/A	0.41	0.67	G/A	0.14	0.34
	22	rs7292425	42,900,747	C/A	0.18	NA	C/A	0.34	NA
	22	rs139107	42,901,313	G/A	0.14	NA	G/A	0.16	NA
	22	rs9614362	42,903,911	G/A	0.30	0.96	G/A	0.13	0.46
	22	rs12483834	42,904,365	C/G	0.02	0.67	C/G	0.16	0.37
	22	rs139116	42,904,875	T/C	0.36	0.28	T/C	0.20	0.02
	22	rs2092351	42,905,737	G/A	0.36	0.31	G/A	0.33	0.18
	22	rs139120	42,905,880	G/A	0.34	0.32	G/A	0.17	0.02
	22	rs878406	42,906,306	A/T	0.02	0.82	A/T	0.19	0.32
	22	rs3747208	42,907,201	A/G	0.10	NA	A/G	0.08	–
	22	rs139123	42,907,418	G/A	0.37	NA	G/A	0.36	NA
	22	rs139124	42,907,465	G/A	0.34	NA	G/A	0.15	NA
	22	rs7287117	42,907,988	G/A	0.02	–	G/A	0.18	NA
	22	rs9306473	42,908,483	G/A	0.002	–	G/A	0	–

Notes:

NA: the failure of passing the quality control procedure (QC).

–: not available due to low minor allele frequency.

Chr: chromosome.

MAF: minor allele frequency.

There are four SNPs that are located in the region of CNV2580 and fifteen SNPs outside the CNV2580 boundaries but inside the gene of SERHL. None of these nineteen SNPs was significantly associated with ALM in the discovery sample, but two SNPs rs139116 and rs139120 were associated with ALM in the replication sample with *p*-values of 0.02 (Table 5).

## Discussion

This is the first CNV–based GWAS for ALM in Caucasians. Two CNVs, CNV1191 and CNV2580, were identified to be associated with ALM.

CNV1191 is located in the gene GIMAP1, which encodes GTPase, IMAP family member 1. GIMAP (GTPase of the immunity–associated protein family) proteins are a family of putative GTPases believed to be regulators of cell death in lymphomyeloid cells. GIMAP1 was the first reported member of this gene family [31]. This gene was involved in the differentiation of T helper (Th) cells of the Th1 lineage, and the related mouse gene has been shown to be critical for the development of the mature B and T lymphocytes [32].

Culturing myotubes from skeletal muscle-biopsies found coordinated reduced expression of five members of the GIMAP family GIMAP1, GIMAP4, GIMAP5, GIMAP6 and GIMAP7, which form a cluster on chromosome 7 and participate in SM cell survival/death [33]. A study in pig skeletal muscle indicated that GIMAP1 was correlated with meat quality and regulation of biological processes involved in the induction of apoptosis [34]. This gene was also involved in regulation of lipid catabolic process, defense response and positive regulation of calcium ion transport [35]. Our findings, combined with the above evidence, support the potential contribution of GIMAP1 to variation in skeletal muscle.

SERHL is a gene coding for a new member of the family of serine hydrolases that is located within peroxisomes [36]. In vivo studies showed that mRNA expression of SERHL increased in response to passive stretch imposed upon skeletal muscle [36].

The association directions of CNV1191 in the discovery and replication studies were different. This inconsistency may be explained by the following reasons. First, genetic variants may have different effects in different populations. A genetic variant may have different allele frequencies among diverse populations because of different evolution histories, which result in different modes of genotype-phenotype association [37]. Second, significant associations are usually found at molecular markers that are in linkage disequilibrium (LD) with causal variant, rather than the causal variant itself. Therefore, the inconsistency in direction could be a result of opposite patterns of LD between the two populations.

Within the two CNVs regions, we did not identify any significant SNPs that were associated with ALM in the discovery sample. A possible explanation is that, different from SNP, CNV is a structural genetic variant that generally covers a larger genomic region and thus CNV may influence phenotypic variation by mechanisms that are different from SNP.

In summary, we identified CNV1191 and CNV2580 that were associated with ALM. The relevant genes, GIMAP1 and SERHL, may play roles in skeletal muscle metabolism. Our findings may provide useful information for molecular functional studies of candidate genes for ALM.

## Supporting Information

Table S1 **Association of normal_deletion and normal_duplication with CNVs.**
(DOCX)
