# Supplementary material for: Genome-Wide Association Study Identified Copy Number Variants Important for Appendicular Lean Mass
Source: PLoS One. 2014 Mar 13;9(3):e89776. doi: 10.1371/journal.pone.0089776 (PMC3953533; doi:10.1371/journal.pone.0089776)
Supplement: Table S1 — Association of normal_deletion and normal_duplication with CNVs. (DOCX) [file pone.0089776.s001.docx]

**SUPPLEMENT**

We performed analyses by including both CNV and covariates in the same model. The results were similar to those from the 2-stage analyses.

Table1. Association of normal_deletion and normal_duplication with CNVs

|  | pair | *β* | SE | *p*-value | Combined-*p*-value |
| --- | --- | --- | --- | --- | --- |
| CNV2580 |  |  |  |  |  |
| Caucasian | normal_deletion | -14.65 | 70.65 | 0.50 | 0.68 |
|  | normal_duplication | -4.32 | 15.46 | 6.53×10^-4^ | 8.81×10^-4^ |
| Chinese | normal_deletion | -14.21 | 150.62 | 0.64 |  |
|  | normal_duplication | -6.41 | 25.46 | 0.13 |  |
| CNV1191 |  |  |  |  |  |
| Caucasian | normal_deletion | -10.65 | 11.25 | 0.44 | 0.11 |
|  | normal_duplication | -8.52 | 76.58 | 2.58×10^-4^ | 1.89×10^-3^ |
| Chinese | normal_deletion | 12.74 | 22.31 | 0.05 |  |
|  | normal_duplication | -9.54 | 93.65 | 0.77 |  |

Notes:

*β*, the standardized regression coefficient, was estimated in kilograms for ALM.

SE: Standard error.
